# Endovascular Applications for the Management of High-Grade Gliomas in the Modern Era

**DOI:** 10.3390/cancers16081594

**Published:** 2024-04-22

**Authors:** Ari D. Kappel, Rohan Jha, Saibaba Guggilapu, William J. Smith, Abdullah H. Feroze, Adam A. Dmytriw, Juan Vicenty-Padilla, Rodolfo E. Alcedo Guardia, Florian A. Gessler, Nirav J. Patel, Rose Du, Alfred P. See, Pier Paolo Peruzzi, Mohammad A. Aziz-Sultan, Joshua D. Bernstock

**Affiliations:** 1 Harvard Medical School, Boston, MA 02115, USA; akappel@bwh.harvard.edu (A.D.K.); g.saibaba13@gmail.com (S.G.); rdu@bwh.harvard.edu (R.D.); pokmeng.see@childrens.harvard.edu (A.P.S.);; 2 Department of Neurosurgery, Brigham and Women’s Hospital, Boston, MA 02115, USA; 3 Department of Neurosurgery, Massachusetts General Hospital, Boston, MA 02114, USA; 4 Neurosurgery Section, School of Medicine University of Puerto Rico, Medical Sciences Campus, San Juan P.O. Box 365067, Puerto Ricorodolfo.alcedo@upr.edu (R.E.A.G.); 5 Department of Neurosurgery, Rostock University Hospital, 18057 Rostock, Germany; 6 Department of Neurosurgery, Boston Children’s Hospital, Boston, MA 02115, USA

**Keywords:** endovascular, high-grade glioma, glioblastoma, interventional neuroradiology, blood–brain barrier, focused ultrasound, future therapies, drug delivery

## Abstract

**Simple Summary:**

This review discusses new treatments for high-grade gliomas (HGGs), aggressive brain tumors that are difficult to treat. It focuses on emerging endovascular therapies and future directions. Endovascular methods use catheters in a minimally invasive manner to deliver drugs to the tumor through blood vessels in the brain while limiting harm to the rest of the body. Super-selective intra-arterial cerebral infusion (SSIACI) is an endovascular technique that aims to deliver treatments directly to the tumor by reaching the closest blood vessels supplying the tumor. Many challenges remain to fully adopting these techniques in clinical practice, including the ability of drugs to cross the blood–brain barrier, and getting the drugs to stay in the tumor for long enough before they are washed out by normal blood in the brain. Focused ultrasound and hyperosmotic disruption are techniques which might help with these challenges. Researchers are investigating new drugs beyond traditional chemotherapies, such as radiation embedded molecules and immune-based therapies. Future directions include monitoring tumors with endovascular sampling; changing drug formulations to stay in the tumor longer; and investigating other treatments, like radioembolization. These endovascular strategies have the potential to improve how HGGs are treated, but more research is needed to ensure they work well and are safe for patients.

**Abstract:**

High-grade gliomas (HGGs) have a poor prognosis and are difficult to treat. This review examines the evolving landscape of endovascular therapies for HGGs. Recent advances in endovascular catheter technology and delivery methods allow for super-selective intra-arterial cerebral infusion (SSIACI) with increasing precision. This treatment modality may offer the ability to deliver anti-tumoral therapies directly to tumor regions while minimizing systemic toxicity. However, challenges persist, including blood–brain barrier (BBB) penetration, hemodynamic complexities, and drug–tumor residence time. Innovative adjunct techniques, such as focused ultrasound (FUS) and hyperosmotic disruption, may facilitate BBB disruption and enhance drug penetration. However, hemodynamic factors that limit drug residence time remain a limitation. Expanding therapeutic options beyond chemotherapy, including radiotherapy and immunobiologics, may motivate future investigations. While preclinical and clinical studies demonstrate moderate efficacy, larger randomized trials are needed to validate the clinical benefits. Additionally, future directions may involve endovascular sampling for peri-tumoral surveillance; changes in drug formulations to prolong residence time; and the exploration of non-pharmaceutical therapies, like radioembolization and photodynamic therapy. Endovascular strategies hold immense potential in reshaping HGG treatment paradigms, offering targeted and minimally invasive approaches. However, overcoming technical challenges and validating clinical efficacy remain paramount for translating these advancements into clinical care.

## 1. Introduction

The annual incidence of high-grade gliomas (HGGs) in the United States is estimated to be 3.19 cases per 100,000 individuals, with approximately 14,000 new cases each year [1]. Glioblastoma (GBM), classified as a World Health Organization (WHO) grade 4 glioma, is the most prevalent malignant primary brain tumor [2]. The prognosis for GBM remains poor, with a median overall survival of 15–18 months with a standard treatment regimen of surgery, radiation, and temozolomide [3], and a 5-year survival rate of only 6.8% [3,4]. Despite substantial advancements in understanding of GBM molecular biology and the identification of new molecular drug targets [5], progress in improving overall survival has remained limited [3]. GBM invariably recurs and disseminates in all patients, and most of these recurrences occur locally [6,7]. Extensive areas of infiltration, necrosis, hemorrhage, and thrombosis within the tumor microenvironment collectively hinder the successful administration of therapeutic drugs, notwithstanding delivery limitations pertaining to the blood–brain barrier (BBB) itself [8]. In response to these challenges, there is a burgeoning interest in local therapies directed to the tumor cavity [9]. The current review focuses on the role of endovascular strategies for local treatment of HGGs to bypass the BBB and prevent local recurrence.

Endovascular treatment for high-grade gliomas (HGGs), including glioblastoma (GBM), was reported as early as the 1950s by Klopp et al. and French et al. [10,11]. The overarching objectives in treating HGG via intra-arterial (IA) chemotherapy is to identify therapeutic agents that can be delivered to the tumor and bypass the blood–brain barrier (BBB) in a reliable and controlled fashion, while minimizing both systemic and neurologic drug toxicity [12]. IA drug administration offers the potential to achieve heightened pharmaceutical drug concentrations within specific tumor regions and increase the likelihood of inducing tumor response. Additionally, IA drug delivery mitigates the often considerable systemic toxicity associated with systemic drug delivery, thereby enabling the exploration of higher chemotherapeutic dosages [13]. These potential benefits have only become more evident with improved endovascular catheter technology allowing for increased specificity in drug delivery with super-selective intra-arterial cerebral infusion (SSIACI) [12]. Despite technological advances and more targeted infusion, studies have failed to show a significant benefit of IA chemotherapy [14]. We conducted an updated review of the literature to highlight recent advancements and to provide an updated view of the two major components and areas of innovation in endovascular therapy for HGGs—the therapy being delivered and the method of delivery.

## 2. Delivery Methods

**Figure 1:**
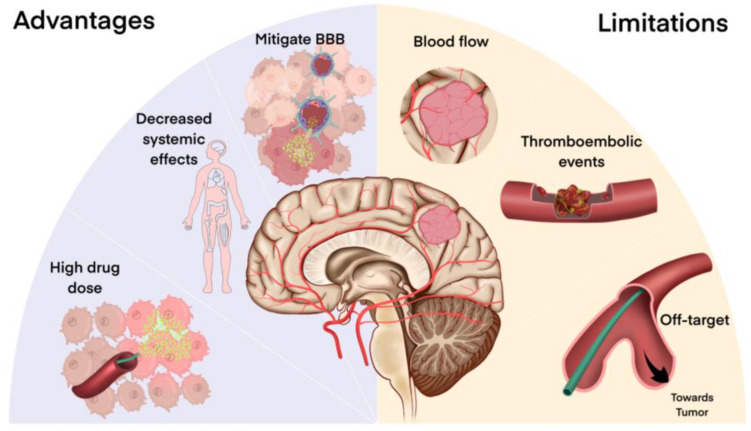
Overview of advantages and limitations of super-selective cerebral arterial infusion (SSIACI) for high-grade gliomas (HGGs). Advantages of this technique include the ability to directly deliver high doses of drugs locally to the tumor bed due to decreased systemic effects with local endovascular drug delivery. Direct endovascular delivery may allow for additional ways to mitigate the BBB through concomitant administration of BBB disruptors such as focused ultrasound. However, off-target effects or thromboembolic events may be complications of endovascular procedures that can cause adverse patient events. Furthermore, normal cerebral blood flow can cause drug washout and decrease the local delivery of therapeutic drugs. The advantages and disadvantages of selective cerebral arterial infusion or endovascular drug delivery to the tumor bed should be considered when designing new therapeutic paradigms.

The development of smaller, more navigable catheters has allowed for the development of increasingly targeted delivery, ushering in the era of selective intra-arterial cerebral injection (SIACI) and super-selective intra-arterial cerebral injection (SSIACI). Advancements in catheter technology have been largely driven by cerebrovascular pathologies and compatibility testing between various chemotherapies, and cell therapies with the catheter materials must be considered and may occasionally pose a challenge [15]. The advantages of super-selective IA delivery include the ability to deliver high drug doses with decreased systemic effects, while limitations include difficulty penetrating the BBB, challenging hemodynamics and pharmacokinetics, off-target effects, and a low drug residence time (Figure 1).

### 2.1. Super-Selective Intra-Arterial Cerebral Infusions

Early non-selective administration of therapeutic agents via IA routes such as carotid or vertebral arteries encountered significant challenges, including profound neurotoxicity, e.g., severe leukoencephalopathy, blindness, and cerebral necrosis [16]. Ototoxicity has been described with IA cisplatin administration, but fewer patients who received IA cisplatin required hearing aids compared to patients who received IV cisplatin [17]. The application of advanced endovascular techniques used in the treatment of cerebrovascular diseases adapted to the super-selective delivery of therapeutic agents for brain tumors has provided technical improvements [18].

Using modern microcatheters, therapeutic agents can be selectively delivered to distal arterial pedicles directly supplying the tumor. Modern neurointerventional techniques involve triaxial endovascular systems [19] with larger bore (6F to 8F) guide catheters and smaller intermediate and microcatheters telescoping from the femoral artery or radial artery to the distal cerebral vasculature [20]. These techniques may help to safely minimize the exposure of normal brain parenchyma to the infused agent by achieving distal access close to the arterial supply of the tumor and avoiding more proximal arterial infusions, such as traditional internal carotid artery or vertebral artery infusions, which may expose more normal brains to neurotoxic chemotherapeutics [21,22]. Drug infusions may be performed manually via hand injections over a period of minutes, with intra-arterial balloons, side ports, or pulsatile injections [21]. This method may restrict the volume of distribution (Vd) of a given agent to the specific pathology and its surrounding vascularized tissues. As a result, drugs may accumulate locally within the tumor, may be delivered at higher doses, and may reduce the systemic levels and subsequent toxicity of a given drug. This approach may be particularly suitable for primary brain malignancies, as they typically exhibit local recurrence without widespread metastasis [12]. SSIACI has demonstrated efficacy particularly in the context of retinoblastoma [23,24] but has also been evaluated for use in GBM [15]. MR perfusion imaging co-registered to cone beam CT to select and monitor IA infusion of chemotherapy has been described as perfusion-guided endovascular super-selective intra-arterial chemotherapy infusion (PG-ESIACI) [25]. This technology allows for optimal vessel selection and the monitoring of chemotherapy delivery through the BBB into the tumor bed, setting a new precedent for locoregional targeting of chemotherapy [21,22].

The uniform delivery of intra-arterially administered drugs to brain tissues is not guaranteed [26,27]. The phenomenon known as “streaming” may result in heterogeneous drug delivery during IA infusion as a result of the flow dynamics, layering of blood in arteries, and insufficient mixing, which may potentially limit treatment effect [22,28]. Strategies to mitigate streaming include large-volume injections exceeding 20% of the background blood flow rate, timing injections to occur during diastole, and using catheters with side ports [22,29]. Spatial dose fractionation algorithms have also emerged, which calculate the necessary agent dose based on vascular perfusion in cerebral vascular territories, ensuring that the dose is proportional to the regional blood flow [26].

In the context of GBM, chemotherapy concentration increases by 20-fold when infused intra-arterially compared to the intravenous (IV) route, translating into a substantial 3–5.5-fold increase in intratumor chemotherapy concentrations due to the highly vascularized nature of most GBM [30]. Moreover, the combination of SSIACI with blood–brain barrier (BBB) disruption for treating malignant gliomas can yield local concentrations of chemotherapeutics in brain tumors over 300 times higher than IV delivery, depending on infusion method, rate, and duration [31]. Although the application of selective or super-selective terminology can vary from different reports, in general, SSIACI achieves high intratumoral drug concentrations when there is low regional blood flow, high regional drug extraction, rapid systemic clearance, and precise tissue concentration (i.e., achieved through pulsed or bolus dosing) [32].

### 2.2. Blood–Brain Barrier Disruptors

The exposure of chemotherapeutic agents to tumor tissue is primarily hindered by the BBB. The BBB represents a complex interplay of endothelial cells, astrocytes, pericytes, basal lamina, extracellular matrix (ECM), smooth muscle cells, and neurons, collectively forming the neurovascular unit (NVU), which governs cerebral blood flow and regulates BBB function [33]. Tight junctions, efflux pumps, and astrocyte podocytes in the BBB limit the passage of ionized molecules with molecular weights that exceed approximately 180 Da [22,34]. This poses a significant challenge to the delivery of various chemotherapeutic agents, and effective drug delivery methods to penetrate or bypass the BBB are required. Therefore, bypassing the BBB is a tantalizing approach to effectively delivering high doses of anti-tumoral drugs locally, while achieving effective doses without the systemic side effects.

There is limited BBB penetration by most anticancer drugs and chemotherapeutics [35]. Temozolomide and topotecan have relatively high CSF/plasma ratios compared to other chemotherapeutics but are still on 20% [36] and 32%, respectively [37]. Most other commonly used chemotherapies have area under the curve (AUC)-CSF-to-AUC-plasma ratios of <5%, including doxorubicin (<5%) [38], cisplatin (3%) [39], carboplatin (2.6%) [39], methotrexate (2.8%) [40], and vincristine (0%) [41].

Hyperosmotic disruption of the BBB is a commonly employed technique for BBB disruption [35]. This strategy involves the infusion of a hypertonic solution, such as mannitol, into the cerebral arteries, thus establishing an osmotic gradient that induces the efflux of water from endothelial cells [42]. Consequently, these cells shrink, and dehydration of endothelial cells interferes with tight junctions, resulting in the heightened permeability of the BBB [43]. Notably, osmotic disruption can enhance the levels of successfully infused chemotherapeutic agents by as much as 90-fold [22].

Focused ultrasound (FUS) has emerged as a promising method for achieving temporary and localized disruption of the BBB in a safe manner. This technique involves the use of low-power FUS, which can provide precise and transient focal disruption of the BBB. The technique employs microbubbles to facilitate the localized and temporary opening of the BBB, thereby allowing drugs to accumulate in the brain parenchyma. During this process, acoustic pressure from a transducer causes microbubbles to be pressed against the endothelial cell wall, inducing temporary and localized disruption of the BBB [44]. FUS has demonstrated its capacity to safely deliver a wide range of therapeutic agents through the BBB in non-human models [45]. These include brain-penetrating nanoparticles, 1,2-bis(2-chloroethyl)-1-nitrosurea, iron oxide magnetic particles conjugated to epirubicin, and gene-based therapy agents [46,47]. Even when drugs manage to penetrate the BBB, they often fail to achieve therapeutically effective local concentrations and frequently cannot attain the effective regional concentrations without inducing unacceptable systemic doses and associated toxicities. The deployment of SSIACI with biplane fluoroscopy combined with extracranial FUS equipment has not been implemented in practice, although extracranial applications of catheter-based ablative ultrasound have been reported with 6Fr devices [48,49]. The combination of an extracranial FUS system which can apply spatial targeting with SSIACI at varying levels of vascular selectivity may lead to clinically relevant BBB disruption in the future.

The combined approach of BBB breakdown and SSIACI could potentially yield significant survival improvements in patients with GBM. When compared to IV infusion, the combination of IA infusion with BBB permeabilization has been reported to result in a remarkable 320-fold-higher drug concentration [30]. This combined strategy holds promise for enhancing the efficacy of drug delivery to brain tumors, particularly in the context of the treatment of HGGs.

### 2.3. Drug–Tumor Residence Time

Increasing the drug–tumor residence time is key to improving outcomes in HGG [50]. A modification of the concept of drug–target residence time first introduced by Copeland and colleagues in 2006 [51], drug–tumor residence time refers to the time a drug spends bound to the tumor in vivo. The concept of drug–tumor residence time avoids the limitations of in vitro assays that measure equilibrium binding metrics and drug-binding affinity and refers to the lifetime of the binary drug–target complex in vivo accounting for absorption, metabolism, and tissue distribution [52]. Furthermore, drug–target residence time in vivo is defined by the inverse of the drug–target dissociation rate constant [52]. Therefore, the longer a drug can remain bound to its target tumor and the lower the dissociation rate constant, the longer the drug–tumor residence time and the longer the anti-tumoral activity of the drug. It is not clear what the optimal drug–tumor residence time is for various drugs and different tumors; however, new drug formulations with nanoparticles and antibody tags, convection enhanced delivery methods, or continuous targeted infusions are strategies to increase the drug–tumor residence time and may be necessary to improve outcomes in HGG [50]. Power et al., found that the delivery of alisertib was effective in the treatment of H3K27M-mutated tumors was only after direct administration over an extended 7-day period [50]. New nanoparticle formulations and delivery methods may help improve drug–tumor residence time [53]. In addition, drug–tumor residence time may modulate the rate of drug metabolism, hence affecting drug efficacy [54]. Given the limitations of the streaming effects on drug residence time with the IA delivery of target therapeutics, new formulations of drugs may be necessary to enhance the efficacy of IA therapeutics for HGG.

### 2.4. Technical Considerations of Endovascular Therapeutics

As discussed so far, failures in effectively achieving therapeutic drug concentrations within the brain via IA therapy include BBB permeability, streaming effects and volume of distribution, pharmacokinetics of CNS drug delivery [30], and endovascular limitations, among others. Here, we discuss these technical considerations, including the role of hemodynamic factors, adjunct imaging techniques, and complication avoidance.

The pharmacokinetics of IA drug delivery is complex. Critical hydrodynamic factors, including background blood flow, injection parameters, and vascular geometry, wield considerable influence over tissue concentrations subsequent to IA drug injections [29]. Hence, a fundamental challenge encountered when transitioning IA treatments to clinical settings is the low predictive ability of incomplete pharmacokinetic modeling. Effective IA drug delivery requires rapid and irreversible uptake during first pass circulation, which can be brief, lasting anywhere from 1 to 10 s in the brain [29,55]. The prevailing approach hinges on the notion that localized injections capable of transiently elevating arterial drug concentrations will elicit the desired pharmacodynamic effects.

However, the dearth of rationalized and methodical selection for IA interventions can lead to suboptimal treatment efficacy or failure. Variability in hydrodynamic factors exist across different vascular territories, different tumors, and even within a given tumor, impacting the delivery and efficacy of chemotherapeutics [56,57]. For example, the success of IA intervention for retinoblastoma likely hinges on targeting a single tumor type, with a few choices of therapeutic agents delivered to a consistent anatomic territory with a few variations in vascular supply [24]. Hence, there is a need to understand the key parameters, such as physiologic and anatomic factors impacting regional blood flow, hydrodynamic facets affecting drug distribution, injection parameters, endothelium–drug interactions, kinetics of BBB uptake and transfer, and site-specific pharmacokinetics [29]. The high resting blood flow of the brain and its susceptibility to embolic injury are paramount limitations to effective IA drug therapy [29].

Advancements in IA drug delivery methods have become pivotal in enhancing therapeutic outcomes. Tumors characterized by low blood flow have demonstrated improved responsiveness to IA chemotherapy [57]; hence, techniques to transiently reduce or arrest blood flow during therapy administration may be useful [32,58]. Approaches include the use of single- or double-balloon catheters designed to isolate proximal and distal arterial flow or anesthetic approaches to decreasing cerebral perfusion. Computational models and preclinical investigations show that, by reducing hydrodynamic stress on injected molecules and extending drug transit time through the cerebral circulation, cerebral hypoperfusion facilitates direct drug delivery to vascular endothelium [27]. Hyperventilation, hypothermia, or deep anesthesia can induce cerebral hypoperfusion [29]. Consequently, flow arrest during IA drug administration yields multiple advantages, including enhanced drug targeting to tumor sites, the attainment of higher cerebral arterial concentrations, a more consistent distribution of drug concentrations in arterial networks, and prolonged transit time, with reduced shear stress and the prevention of drug binding to blood proteins or cellular elements [59].

The integration of magnetic resonance imaging (MRI) into the guidance of IA infusions may be an advantageous approach, as evidenced by recent preclinical and clinical studies [60]. Real-time MRI-guided infusion may allow for precise targeting and enhance the assessment of tumor uptake to the intended target area. Pioneering work by Zawadzki et al. demonstrated the technical feasibility and safety of performing targeted IA cerebral infusions under real-time MRI guidance [61]. This approach is particularly valuable due to the variable vascularity observed in GBM. Real-time MRI guidance during microcatheter may provide crucial quantification of the overlap between the transcatheter perfusion territory and the enhancing mass, enhancing precision and efficacy [25]. Incorporating cone-beam computed tomography (CBCT) into the planning of IA injections and determining the area of infusion further enhance specificity. CBCT facilitates the generation of perfusion maps, improves the accuracy of IA drug delivery, minimizes the exposure of healthy brain parenchyma, and enhances the therapeutic ratio [25].

Complications, including vessel rupture or stroke, although rare, are potentially catastrophic. In some cases, super-selective IA drug delivery may not be technically feasible or may impart an unacceptably high risk to the patient. In the case of tumors supplied by small distal branches that are difficult to access, patients with variant anatomy, or specific technical challenges, the possibility of neurologic complications may be increased, and the precise vascular supply may be difficult to isolate or access. Radiated and recurrent tumors may recruit novel collateral vascular supply, lack discernible angiographic tumor blush, and may be difficult to target endovascularly.

## 3. Therapies

**Figure 2:**
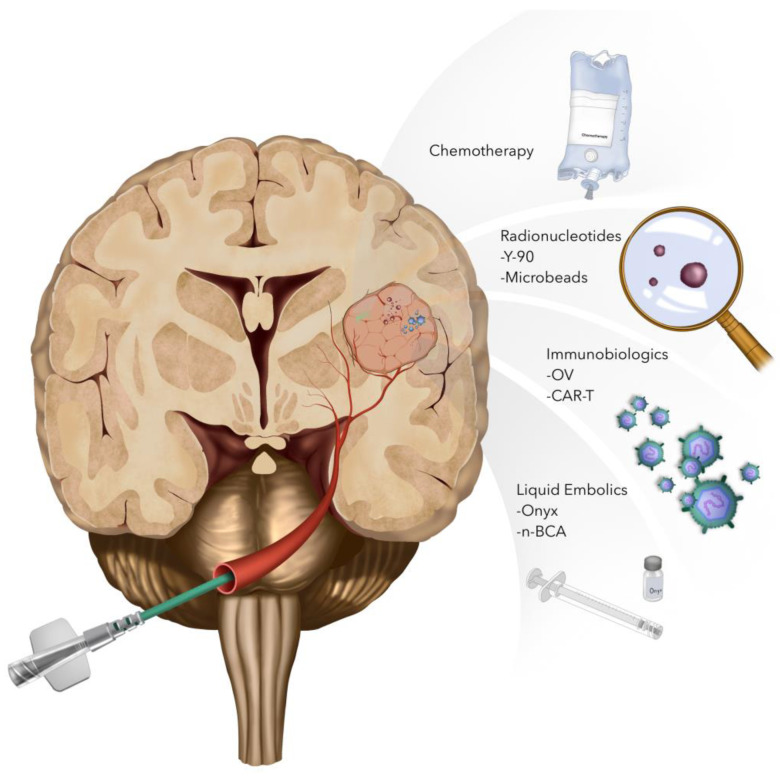
Multiple therapeutic drugs and treatments may be delivered endovascularly. Super-selective cerebral arterial infusion of chemotherapeutics, such as methotrexate, vincristine, diaziquone, etoposide, idarubicin, and bevacizumab, has been trialed for high-grade gliomas. Radionuclides, such as radioactive yttrium-90, can be delivered endovascularly on microbeads or with PVA microbubbles. Oncolytic viruses and CAR-T cells may also be delivered directly to the tumor or tumor bed endovascularly. Traditional endovascular techniques, including embolization with liquid embolics, remains a potential adjuvant option to increase tumor residence time of delivered drugs or decrease tumoral blood supply.

The intra-arterial delivery and SSIACI of multiple chemotherapeutics, radiotherapies, and immunobiological therapies have been studied for the treatment of HGGs (Figure 2). SSIACI may decrease the systemic effects of highly toxic chemotherapeutics or radiotherapies and may increase the local dose that can be delivered to the target tissue. However, many challenges remain. Here, we review specific drugs and targets for the IA drug delivery in the management of HGGs.

### 3.1. Chemotherapeutics

To date, a range of non-selective IA chemotherapeutic agents, including nitrosourea derivatives, platinum analogs, methotrexate, and vincristine, have been employed in the treatment of malignant gliomas [27]. In addition, various IA chemotherapeutics, such as diaziquone, etoposide, and idarubicin, have also been tested [21]. Historically, nitrosourea derivatives have been associated with severe neurotoxicity [16], whereas platinum analogs have demonstrated fewer cerebral side effects [12]. Temozolomide, a key component of the gold-standard Stupp protocol, has been found to have limited utility in IA infusion [62], as it requires repetitive cycles to bypass the inherent resistance of glioblastoma stem cells to the drug [63,64]. One small phase I study of IA bevacizumab after BBB disruption followed by IV bevacizumab showed a good safety profile with modest but promising results [64].

At present, there are several ongoing clinical trials investigating chemotherapy for IA infusion, of which four utilize bevacizumab (Table 1). One study is investigating cetuximab, an epidermal growth factor receptor inhibitor found to be well tolerated at high doses [31]. A phase 0 study testing the blood–brain barrier (BBB) permeability of temsirolimus, an FDA-approved mTOR inhibitor for renal cell carcinoma, is ongoing. Uluc et al., published a large retrospective analysis of 4939 IA chemotherapy deliveries in 436 patients with various brain tumors (primary central nervous system lymphoma (26.4%), glioblastoma (18.1%), and oligodendroglioma (14.7%)), with and without blood–brain barrier disruption, using a 25% mannitol infusion, with patients most often receiving a methotrexate injection through the ICA [65]. They found a higher incidence of seizures in BBB-disruption cases (5.32% vs. 0.18%, *p* < 0.001), but no significant difference was seen in major complications with a rate of ≤1% in both groups [65]. Uluc et al., demonstrated the relative safety and tolerability of IA chemotherapy with enhanced drug delivery to the brain tumor and the surrounding parenchyma [65]. Lim et al., reported the first rat model of GBM amenable to the testing of bevacizumab, carboplatin, and irinotecan through a left internal carotid artery (ICA) injection [66]. This model allows for further testing of novel chemotherapeutics and other therapies via endovascular delivery. A recent case series of 12 patients from Chongqing University in China investigated the SSIACI of teniposide, an inhibitor of DNA repair which binds to topoisomerase II [67], and found encouraging early results [68]. Endovascular delivery of chemotherapy may have a promising safety profile, but BBB penetration, tumor residence time, and the ideal chemotherapy and its most effective dose are problems that remain to be solved in this space. The SSIACI of bevacizumab has been the most widely studied chemotherapeutic, but ongoing trials of bevacizumab, as well as immunotherapeutics, such as cetuximab, will help to define the future role of SSIACI in the armamentarium of management of HGGs.

### 3.2. Radiotherapy

Targeted radionuclide therapy with β-emitting radionuclides, including Yttrium-90 (Y90) and iodine-131; α-particles; and auger electron emitters have been investigated for their therapeutic efficacy in HGGs [69]. β-emitting radionuclides have been most extensively studied [70]; however, α-particles, such as actinium-225, astatine-211, and bismuth-213, may be useful for preventing micro-metastases or treating residual tumors [69,71], and auger electron emitters, such as [^125^I]5-Iodo-2′-deoxyuridine ([^125^I]I-UdR), have been demonstrated to increase therapeutic effects when combined with temozolomide with or without methotrexate [72]. The IA delivery of Y90 microspheres has been explored as an endovascular brachytherapy treatment for GBM. In a canine model of GBM, a microcatheter technique was employed to selectively infuse Y90 glass microspheres intra-arterially [73]. At the one-month follow-up after therapy, animals displayed a substantial reduction in mass volume, ranging from 24% to an impressive 94% [73]. Y90 glass microspheres were FDA approved in the United States for treatment of hepatocellular carcinoma in 2021 [74], but they are not approved for intracranial use or currently used in clinical settings other than for research. The delivery strategy for radioactive lanthanides, such as Y90, in the context of GBM therapy, involves addressing the formulation of suitable delivery carriers. These carriers should have a high loading capacity for radiotherapeutic agent, be compatible with endovascular techniques and microcatheters, and have the ability to selectively accumulate in the tumor [75]. One approach is ultrasonic microbubbles with polyvinyl alcohol (PVA) shells to load yttrium into a substrate capable of being delivered by a microcatheter as an endovascular radiopharmaceutical infusion [76]. Achieving selective targeting of yttrium-loaded microbubbles (MBs) on glioblastoma-associated tumor endothelial cells may be attainable through biorecognition mechanisms. Specifically, the overexpressed αVβ3 integrin can interact with the ligand Cyclo(Arg-Gly-Asp-D-Phe-Lys) present on the PVA microbubble surface, facilitating precise delivery to the tumor site [76]. The main limitation of radiopharmaceuticals includes limited BBB penetration properties of conjugated ligands, heterogeneous antigen expression limiting target-mediated therapy, and translatability from preclinical models [69]. However, the advantages of IA delivery may help mitigate some of these issues. Future studies on material science, radiotherapeutic loading, BBB penetration, and tumor penetration and targeting are needed to create more effective radiotherapeutic drugs that can be delivered endovascularly.

### 3.3. Immunobiologics

Immunobiologics represents a novel area of cancer therapy and oncology research focused on harnessing and modifying the human immune system to target cancer cells. Within this category, two therapies, oncolytic viruses and chimeric antigen receptor T-cell therapy (CAR-T), have been explored for intra-arterial delivery. Oncolytic viruses (OVs) are a burgeoning area of research in the treatment of GBM [77]. Briefly, OVs are viruses designed to replicate specifically in tumor cells, inducing oncolysis and developing adaptive immunity [15,78]. Adenovirus has been extensively studied for the treatment of GBM, with adenovirus DNX-2401 (formerly known as Delta-24-RGD) used in the first clinical study demonstrating direct oncolysis and providing evidence for a viral-induced anti-glioma immune response [79,80]. The use of mesenchymal stem cells (MSCs) for the delivery of DNX-2401 (MSC-DNX-2401) and their ability to target GBMs when delivered IA is now being explored [15]. DNX-240 has shown success in reducing tumor size and prolonging survival in some GBM patients when administered via intratumoral injections and is currently in a phase I trial for endovascular delivery for recurrent GBM [80,81].

Similarly, CAR-T therapy has demonstrated notable clinical efficacy in diverse solid tumors [82]. Although the data remain limited regarding CAR-T cell therapy delivery for malignant gliomas, ongoing preclinical and interventional clinical investigations suggest enhanced effectiveness through locoregional delivery. Moreover, researchers have made noteworthy progress in the SSIACI of activated T cells in rabbit models, showcasing safe infusion without catastrophic embolic–ischemic adverse events [83].

Kan et al., developed a rabbit model of GBM that demonstrated the safety of distal ICA delivery of 2 mL of MSC-DNX-2401 in 25 rabbits with histologic evidence of homing to the tumor at 24 h post-injection [84]. Several new trials of direct intratumoral administration of OVs are ongoing, including a phase II trial of G47-delta an oncolytic herpes virus, adjuvant research combining adenovirus OVs with therapies such as pembrolizumab or CAR-T therapy, and preclinical work in novel OV discovery [85,86,87,88,89]. This is of particular relevance, as the development of efficacious intra-arterial delivery of CAR-T cells would represent the frontline of locoregional CAR-T therapy for GBM [90]. As research continues to progress in immunobiologics therapy for GBM, the novel rabbit GBM model developed by Srinivasan et al., will play a pivotal role in the accelerated bench-to-bedside progression of new therapies.

### 3.4. Liquid Embolics

Traditional pre-operative embolization with liquid embolic agents [91] has not been typically used in the management of intrinsic HGGs. Some groups have experimented with endoscopic surgery for HGGs with adjuvant pre-operative tumor embolization in order to reduce blood loss and improve the safety of minimally invasive surgery [92]. Although more commonly utilized in extra-axial brain and head and neck tumors, including meningioma and paraganglioma, arterial embolization may play a role in the multi-modal management of HGGs in select, highly vascular cases with deep arterial supply that may be difficult to access surgically. Future experiments may consider liquid embolics after drug delivery as a potential method to mitigate hemodynamic effects, decrease drug washout, and increase tumor residence time, or as an adjuvant method of decreasing tumoral blood supply.

## 4. Future Directions

Despite the first description of IA drug delivery for HGG in the 1950s [10,11], adjunctive endovascular treatments remain experimental. Although super-selective IA cerebral infusions (SSIACIs) offer a tantalizing way to increase the effective drug dose delivered to the target tumor tissue directly with decreased systemic effects, many challenges remain, including the limited ability to penetrate the BBB, challenging pharmacokinetics and hemodynamics, off-target effects, and low drug residence time. Future research targeting these limitations may help bring SSIACI into the future multi-modal treatment regimen for difficult-to-manage HGGs.

### 4.1. Endovascular Sampling of Peri-Tumoral Vasculature

Super-selective catheterization allows for selective sampling from the vessels surrounding a GBM resection cavity. With therapies that modulate the BBB, the ability to obtain highly localized CSF, interstitial fluid, and blood from around the tumor may allow for improved surveillance of recurrence, particularly in patients who have radiation necrosis that mimics recurrence radiographically. Technologies such as liquid tumor biopsy are already being developed and could be paired with endovascular sampling to improve diagnostic yield [93,94].

### 4.2. Drug Formulations

Low drug residence times due to high cerebral blood flow and challenging pharmacokinetics remains a limitation of IA drug delivery. Nanoparticle-encapsulated talazoparib injected intrathecally has shown promise for increasing penetration and therapeutic index [95]. Similar encapsulated drug formulations may be translated to endovascular interventions to improve tumor targeting and increasing treatment efficacy. Rainov et al., used a herpes simplex virus vector and monocrystalline iron oxide nanoparticles in conjunction with bradykinin-mediated blood–tumor barrier disruption to target gliosarcomas in rats and demonstrated improved uptake and viral-mediated gene delivery [96]. Ligand conjugation is a strategy that actively targets endothelial cell receptors such as transferrin receptors, insulin receptors, or lipoprotein receptors that may improve the ability to cross the BBB and target specific brain regions [97,98]. Additionally, several novel nanoparticle systems for the delivery of drugs to brain tumors have been developed but have not been trialed in IA delivery systems [99]. Together with improved endovascular delivery techniques, these new nanoparticle formulations may improve brain-tumor treatments moving forward. Applications of advances in materials science, nanoparticles, and novel drug formulations that improve BBB crossing and/or increase drug–tumor residence time may improve the efficacy of super-selective IA drug delivery in the future [100,101].

### 4.3. Endovascular Delivery of Non-Pharmaceutical Therapies

One unique advantage of endovascular drug delivery is the option to go beyond medication delivery and offer endovascular embolization as well. Targeted radioembolization may be a potential pre-operative adjuvant therapy to help reduce microscopic seeding and decrease local recurrence [76]. Intra-operative photodynamic exposure to a tumor premedicated with 5-ALA may induce a thermally mediated and immunologically mediate tumor ablation [102,103]. Endovascular catheters could potentially provide a light source for minimally invasive, highly selective delivery to the tumor. Further research into photodynamic endovascular therapy could provide promise for localized GBM ablation.

## 5. Conclusions

The potential benefits of endovascular intervention for GBM treatment are plentiful, underscored most prominently with the promise of flexible, multimodal, and targeted chemotherapeutic delivery while minimizing systemic toxicity. Several studies have proven the safety of SSIACI in its ability to deliver chemotherapeutics. Promising phase I and phase II studies have demonstrated moderate efficacy. Nonetheless, large, randomized phase III trials have been limited by high costs and patient-recruitment challenges and limit the interpretation of SSIACI efficacy. Nevertheless, with a better understanding of GBM morphology, improved chemotherapeutics, BBB disruption formulations, and refinement of clinical techniques, endovascular approaches hold high promise for improving patient outcomes while minimizing chemotoxicity.

## Figures and Tables

**Table 1:** Ongoing trials of super-selective intra-arterial cerebral infusion are listed, along with the treatment used, the study type, patient cohort, and outcomes measured.

Study	Treatment	Study Type	Patient Cohort	Outcomes
“NCT02285959 Super-Selective Intraarterial Intracranial Infusion of Bevacizumab (Avastin) for Glioblastoma Multiforme”	Bevacizumab repeated every 3 weeks	Phase I single-arm prospective study	Recurrent GBM after resection	Primary: Adverse events Secondary: Tumor response
“NCT02861898 Super-Selective Intra-Arterial Repeated Infusion of Cetuximab for the Treatment of Newly Diagnosed Glioblastoma”	Cetuximab and Mannitol for 3 doses q3 months	Phase I/II single-arm prospective study	Newly diagnosed GBM	Primary: Progression-free survival at 6 months and overall survival at 2 years Secondary: Composite overall response rate and toxicity by CTCAE
“NCT05271240 Repeated Superselective Intraarterial Cerebral Infusion (SIACI) of Bevacizumab with Temozolomide and Radiation Compared to Temozolomide and Radiation Alone in Newly Diagnosed GBM”	Bevacizumab and mannitol + Temozolomide and XRT 3 doses q3 months	Phase III randomized control trial	Newly diagnosed GBM	Primary: Overall survival Secondary: Progression-free survival
“NCT01269853 Repeated Super-Selective Intraarterial Cerebral Infusion of Bevacizumab (Avastin) for Treatment of Relapsed GBM and AA”	Bevacizumab and mannitol q2 week +/− IV bevacizumab	Phase I/II two-arm non-randomized prospective study	Recurrent GBM and anaplastic astrocytoma	Primary: Composite overall response; progression-free survival and overall survival at 6 months Secondary: Toxicity
“NCT05773326 Superselective Intra-Arterial Cerebral Infusion of Temsirolimus in HGG”	Temsirolimus single infusion	Phase 0 single-arm prospective study	Newly diagnosed GBM pre-operatively	Primary: Total and unbound temsirolimus in tumor tissue Secondary: Quantification of pS6 positive cells
“NCT02800486 Super Selective Intra-Arterial Repeated Infusion of Cetuximab (Erbitux) with Reirradiation for Treatment of Relapsed/Refractory GBM, AA, and AOA”	Cetuximab with mannitol and radiation	Phase II prospective study	Relapsed/refractory GBM, AA, AOA	Primary: PFS at 6 months and OS at 2 years Secondary: CORR and toxicity via CTCAE
“NCT05956821 Treatment of Relapsed/Refractory Intracranial Glioma in Patients Under 22 Years of Age”	Cetuximab and bevacizumab q1 month for 1 year	Phase I/II prospective study	Recurrent GBM < 22 years old	Primary: Adverse events, CORR, and PFS and OS at 1 year
“NCT03896568 MSC-DNX-2401 in Treating Patients with Recurrent High-Grade Glioma”	MSC-DNX-2401 oncolytic adenovirus 1–2 infusions 2 weeks pre-op + intramural injection	Phase I prospective study	Recurrent GBM	Primary: Max tolerated dose and adverse events Secondary: Tumor response, time to progression, virus replication in tumor, virus shedding, and adenoviral antibodies
